# Single-Cell Electrical Phenotyping Enabling the Classification of Mouse Tumor Samples

**DOI:** 10.1038/srep19487

**Published:** 2016-01-14

**Authors:** Yang Zhao, Mei Jiang, Deyong Chen, Xiaoting Zhao, Chengcheng Xue, Rui Hao, Wentao Yue, Junbo Wang, Jian Chen

**Affiliations:** 1 State Key Laboratory of Transducer Technology, Institute of Electronics, Chinese Academy of Sciences, Beijing, P.R. China, 100190; 2 Department of Cellular and Molecular Biology, Beijing Chest Hospital, Capital Medical University, Beijing, P.R. China, 101149

## Abstract

Single-cell electrical phenotyping (e.g., specific membrane capacitance (C_m_) and cytoplasm conductivity (σ_p_)) has long been regarded as potential label-free biophysical markers in tumor status evaluation. However, previous studies only reported the differentiation of tumor cell lines without classifying real tumor samples using cellular electrical properties. In this study, two types of mouse tumor models were constructed by injecting two types of tumor cell lines (A549 and H1299), respectively. Then tumor portions were retrieved for immunohistochemistry studies and single-cell electrical phenotyping based on home-developed microfluidic platforms. Immunohistochemistry results of tumor samples confirmed the adenocarcinoma and large-cell carcinoma characteristics for A549 and H1299 based tumor samples, respectively. Meanwhile, cellular C_m_ and σ_p_ were characterized as 2.25 ± 0.50 μF/cm^2^ and 0.96 ± 0.20 S/m for A549 based tumor samples (n_cell_ = 1336, Mouse I, II, III) and 1.76 ± 0.54 μF/cm^2^ and 1.35 ± 0.28 S/m for H1299 based tumor samples (n_cell_ = 1442, Mouse IV, V, VI). Significant differences in C_m_ and σ_p_ were observed between these two types of tumor samples, validating the feasibility of using C_m_ and σ_p_ for mouse tumor classification.

Single-cell electrical phenotyping (e.g., the quantification of specific membrane capacitance (C_m_) and cytoplasm conductivity (σ_p_)) is important for understanding cellular functions and status^1^,^2^, enabling the classification of tumor cells^3^,^4^,^5^,^6^ , stem cells^7^,^8^,^9^,^10^ and blood cells^11^,^12^,^13^,^14^,^15^,^16^.

In the field of electrical phenotyping of tumor cells, there are mainly two conventional techniques, which are dielectrophoresis and electrorotation^17^,^18^. In dielectrophoresis, cells attached to dielectrophoretic electrodes at specific frequencies were counted, and the number of attached cells was then translated to intrinsic electrical properties^19^. Based on this technique, electrical property variations of tumor cells with different malignant levels (e.g., normal keratinocytes versus oral squamous cell carcinoma cell lines^4^,^20^,^21^) and different anti-drug capabilities (e.g., K562 cells vs. their multidrug resistant derivatives^3^, MCF 7 cells vs. their multidrug resistant derivatives^5^) were reported. However, this technique can only provide electrical properties based on batch testing and cannot quantify electrical properties at the single cell level.

In electrorotation, a rotating electric field is applied to rotate a suspended cell as a result of Maxwell-Wanger polarization^22^,^23^. Based on this technique, electrical property variations of tumor cells with different anti-drug capabilities (e.g., K562 cells vs. their multidrug resistant derivatives^24^) were reported. However, in electrorotation, cell manipulation and positioning in the rotating electric field is time consuming and labor intensive.

With the development of microfluidics featured with typical dimensions in the range of 1–100 μm^25^,^26^, micro electrical impedance spectroscopy was proposed for cell classification at the single cell level^27^,^28^,^29^,^30^. Frazier *et al*. sucked cells on top of electrodes and classified breast^31^ as well as head and neck cells^32^ with different malignant levels. Sun *et al*. aspirated single cells through a microfluidics based constriction channel (cross sectional area smaller than biological cells) for cellular impedance measurement, enabling classification of breast tumor cells and their multiple drug resistant counterparts^33^. However, these classifications can only quantify size-dependent electrical parameters which are heavily dependent on experimental conditions, which cannot indicate intrinsic cellular electrical properties (C_m_ and σ_p_).

Recently, we proposed a microfluidic platform enabling the high-throughput quantification of C_m_ and σ_p_ by modeling the cellular travelling process within the constriction design^34^,^35^. Using this system, electrical variations of paired high- and low-metastatic carcinoma strains, as well as tumor cells with single oncogenes under regulation were reported based on measurement results from hundreds of cells^6^. However, previous studies only reported the differentiation of tumor cell lines without classifying real tumor samples using C_m_ and σ_p_.

To address this issue, in this study, we conducted single-cell electrical phenotyping of two types of mouse tumor samples, which were constructed by injecting two types of tumor cell lines, respectively. Then tumor portions were retrieved for single-cell electrical phenotyping based on the home-developed microfluidic platforms. Significant differences in C_m_ and σ_p_ were observed between these two types of tumor samples, validating the feasibility of using C_m_ and σ_p_ for mouse tumor classification.

## Materials and Methods

### Materials

Materials used for isolation of solid tumor samples include CytoSelect™ Clonogenic Tumor Cell Isolation Kit (Cell Biolabs, Inc. San Diego, CA, USA) and collagenase II (Sigma, St. Louis, MO, USA). All cell-culture reagents were purchased from Life Technologies Corporation (Carlsbad, CA, USA) unless otherwise specified. The materials used for the fabrication of microfluidic devices were SU-8 photoresist (MicroChem Corp, Newton, MA, USA) and 184 silicone elastomer (Dow Corning Corp., Midland, MI, USA).

### Mouse Tumor Formation, Retrieval, and Colony Formation

Female BALB/c-nude mice were obtained from the Experimental Animal Center of NICPBP (China’s National Institute for the Control of Pharmaceutical and Biologic Products). Mice were maintained in a pathogen-free environment, with temperature and humidity constant. All procedures involving animals were approved by the Capital Medical University Animal Care and Use Committee and were carried out in accordance with the approved guidelines. Xenograft assays were performed with 3 animals for each cohort and H1299 or A549 cells (2 × 10^6^ in 0.2 mL PBS) were injected subcutaneously. After seven weeks, mice were sacrificed and the tumor portions were excised and retrieved.

Tumors from mice were formalin-fixed and paraffin-embedded. Paraffin sections were dewaxed in xylene and rehydrated with xylene/alcohol (1:1), 100% alcohol, 95% alcohol, 90% alcohol, 80% alcohol, 70% alcohol and 50% alcohol successively. After stained with hematoxylin and eosin, the sections were hydrated with 95% alcohol, 100% alcohol and xylene successively. Images were taken in a Nikon microscope through a 20 × objective.

For the isolation of solid tumor samples, CytoSelect™ Clonogenic Tumor Cell Isolation Kit (Cell Biolabs) was used. Briefly, xenograft tumors were minced with a razor blade and washed several times in PBS with high doses of penicillin/streptomycin to avoid contamination. Tissue dissociation was carried out by enzymatic digestion (0.8 mg/ml collagenase II, Sigma) at 37 °C for 4 hours to allow complete cell dissociation with pipetting every 1 hour. Cells were filtered through 40 μm sterile filter and adjusted to 5 × 10^6 ^cells/mL. Then cells were incubated 14 days in a proprietary semisolid agar media. Once these cells formed colonies, they were retrieved from the agar media and separated from the single cells by using a 70 μm sterile filter. The viable cells in these colonies were then dissociated into individual cells and seeded in 6-well plates for single-cell electrical phenotyping.

### Microfluidic Device Fabrication, Operation and Data Analysis

The two-layer PDMS device (constriction channel cross-section area of 10 μm × 10 μm) was replicated from a double-layer SU-8 mold, with detailed fabrication procedures described in previous publications^6^,^36^. Briefly, the first layer of SU-8 5 was used to form the constriction channel (10 μm) and the second layer of SU-8 25 was used to form the cell loading channel (25 μm). PDMS prepolymer and curing agent were mixed, degassed, poured on channel masters and baked in an oven. PDMS channels were then peeled from the SU-8 masters with reservoir holes punched through and bonded to a glass slide.

The whole detailed operation process and data processing was described in previous publications^6^,^36^. Briefly, the cell samples were pipetted to the entrance of the cell loading channel of the microfluidic device where a negative pressure at 1 kPa was applied to aspirate cells continuously through the constriction channel with two-frequency impedance data (1 kHz + 100 kHz) and images recorded (sampling rate: 20 points per sec, experimental duration: 750 sec as one experiment, throughput: ~1 cell per sec). Raw impedance data were translated to impedance data with cells at 1 kHz and 100 kHz, which were used to evaluate the sealing properties of deformed cells with constriction channel walls and equivalent cellular membrane capacitance as well as cytoplasm resistance, respectively. By combining cell elongation length during its traveling process within the constriction channel based on image processing, these impedance data were further translated to C_m_ and σ_p_.

## Results and Discussion

Currently, the golden-standard approach for tumor classification is the immunohistochemistry of tumor samples where morphology data obtained from hematoxylin and eosin staining were used for tumor status evaluation. However, this is a qualitative approach which requests extensive personnel training to make sound decisions. In this study, we explored the feasibility of using electrical parameters including C_m_ and σ_p_ to classify tumor samples, which may provide a quantitative approach for cellular status evaluation.

**Figure 1:**
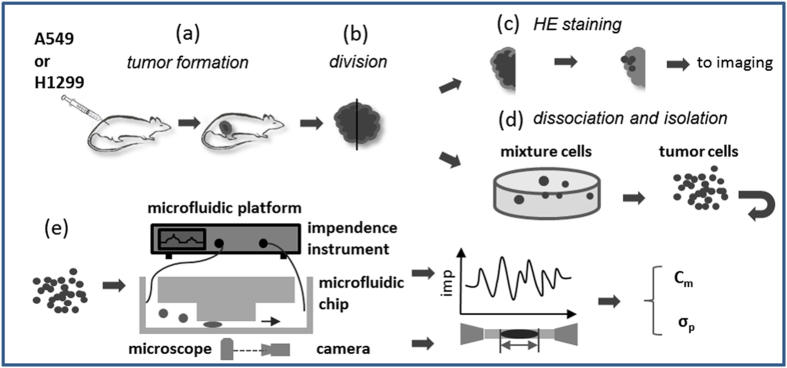
Schematic of this study. (**a**) mouse tumor formation (subcutaneous injection of lung tumor cells into nude mice). (**b**) tumor sample retrieval and division into two portions. (**c**) hematoxylin and eosin staining. (**d**) sample dissociation and seeding in agar media for purification (removal of fibroblast-like cells). (**e**) electrical property characterization of single tumor cells with C_m_ and σ_p_ quantified.

As shown in Fig. 1, in this study, initially, two human lung tumor cell lines (A549 and H1299) were injected subcutaneously into nude mice, respectively, to form solid tumors (see Fig. 1(a)). Then tumor samples were excised and divided into two portions (see Fig. 1(b)). For the immunohistochemistry assay, tumor samples were formalin-fixed and stained by hematoxylin and eosin (see Fig. 1(c)). In the meanwhile, xenograft tumor samples were dissociated by enzymatic digestion and seeded in agar media to remove fibroblast like cells (see Fig. 1(d)). Purified tumor clones were then retrieved from agar media to form suspended single cells, which were then flushed into the constriction channel based microfluidic platform with C_m_ and σ_p_ quantified (see Fig. 1(e)).

**Figure 2:**
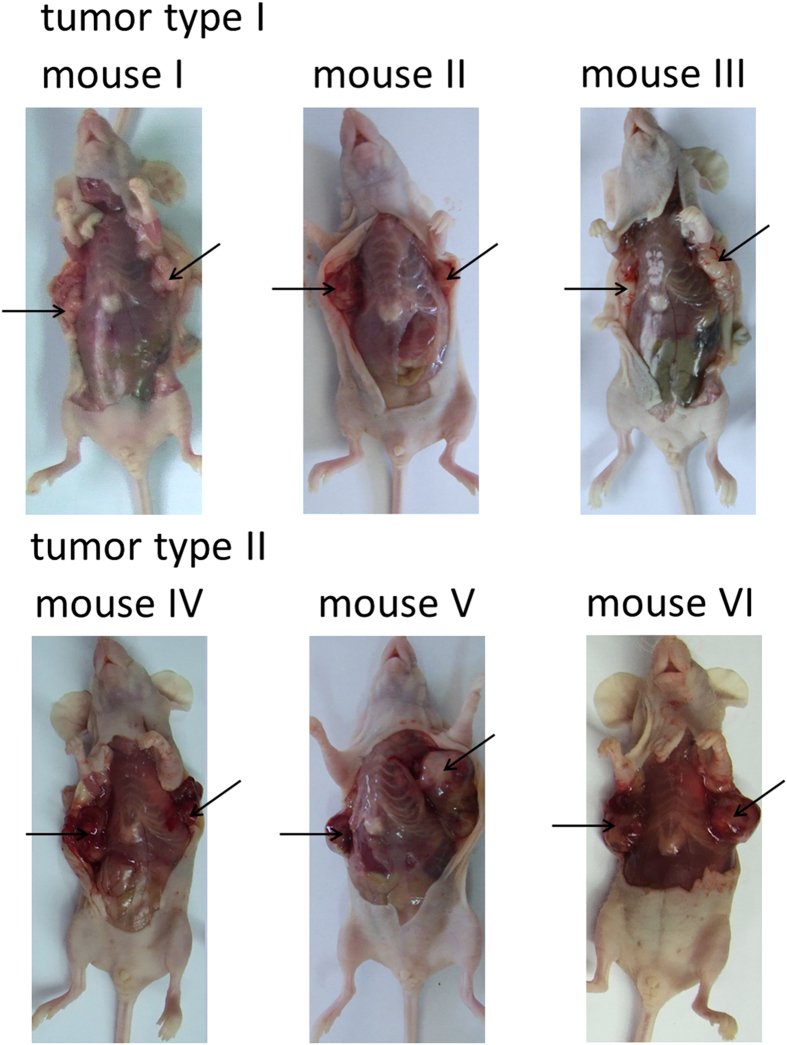
Images of sacrificed mice with the tumor portions marked by arrows. A549 (tumor type I) and H1299 (Tumor Type II) cells were injected in the flank of 6-week-old female BALB/c-nude mice subcutaneously. Tumors were noticed after 2–3 weeks of tumor cell injection and mice were sacrificed in 7 weeks with the tumor portions retrieved.

After the injection of lung tumor cell lines into nude mice subcutaneously, tumor formation was noticed within two weeks and mice appeared weight loss seven weeks later. Then six mice in total (three mice injected with A549 cells and three mice injected with H1299 cells) were sacrificed with tumor samples retrieved. As shown in Fig. 2, three mice injected with A549 cells and three mice injected with H1299 cells were sacrificed with tumor portions marked with arrows.

**Figure 3:**
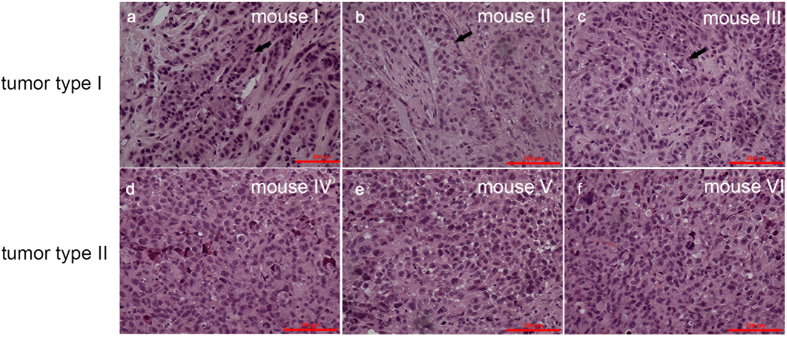
Immunohistochemistry results of xenograft tumor samples. (**a–c**) Tumor samples from three mice injected with A549 cells are featured with adenocarcinoma characteristics (glandular cavities) since A549 is an adenocarcinoma cell line. (**d–f**) Tumor samples from three mice injected with H1299 cells are featured with large cell carcinoma characteristics (polygonal-shaped cells) since H1299 is a large cell neuroendocrine cell line.

For each retrieved tumor sample, it was divided into two portions. One portion of the tumor sample was formalin-fixed and paraffin-embedded. After stained with hematoxylin and eosin, the images were taken for tumor type classification. Figure 3(a–f) represent tumor samples from three mice injected with A549 cells or H1299 cells, respectively. Significant differences in the hematoxylin and eosin staining were located, due to the pathological difference of A549 and H1299 cells. A549 is an adenocarcinoma cell line and therefore adenocarcinoma characteristics (i.e., glandular cavities) were observed in corresponding xenograft tumor samples (see Fig. 3(a–c)) while H1299 is a large cell neuroendocrine cell line and therefore large cell carcinoma characteristics (i.e., polygonal-shaped cells) were located in corresponding xenograft tumor samples (see Fig. 3(d–f)). The consistency between the injected tumor cell types and morphologies of xenograft tumor samples confirmed the successful formation of tumor samples. Note that although a significant difference in cellular morphologies between these two types of tumors can be obtained, this approach is qualitative and cannot provide quantitative data.

**Figure 4:**
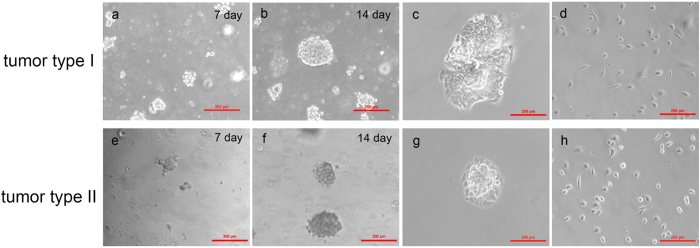
Time-sequence tumor colony formation, tumor cluster retrieve and seeded in culture plates. Tumor colony formation for A549 based tumor samples (**a**,**b**) and H1299 based tumor samples (**e**,**f**). (**c**,**g**) show the retrieved tumor clusters collected from A549 and H1299 based tumor samples, respectively. These tumor colonies were further dissociated into individual cells and seeded in 6-well plates for single-cell electrical phenotyping (**d**,**h**).

The second portion for each retrieved tumor sample was dissociated into individual cells and cultured in semisolid agar media to form tumor colonies. This approach is a well-established method, capable of forming pure tumor colonies by removing impuries such as tumor-associated fibroblasts which cannot effectively proliferate in the environment of semisolid agar media (CytoSelect™ Clonogenic Tumor Cell Isolation Kit, Cell Biolabs). As shown in Fig. 4, it is a time-sequence proliferation of tumor cells to form the tumor colonies (A549 based tumor colony formation for Fig. 4(a,b) and H1299 based tumor colony formation for Fig. 4(e,f)). Figure 4(c,g) show the retrivied tumor clusters from A549 and H1299 based tumor samples, respectively. These tumor colonies were further dissociated into individual cells and seeded in 6-well plates for single-cell electrical phenotyping (see Fig. 4(d,h)).

Single-cell electrical phenotyping was realized by a home-developed microfluidic platform where single-cell suspensions were pipetted to the entrance of the microfluidic device and a negative pressure was applied to aspirate cells continuously through the constriction channel (a channel with a cross-sectional area smaller than a cell) with two-frequency impedance data (1 kHz + 100 kHz) and images recorded. Note that 1 kHz impedance data was used to evaluate the cell-channel leakage and 100 kHz impedance data was used to quantify membrane capacitance and cytoplasm resistance.

**Figure 5:**
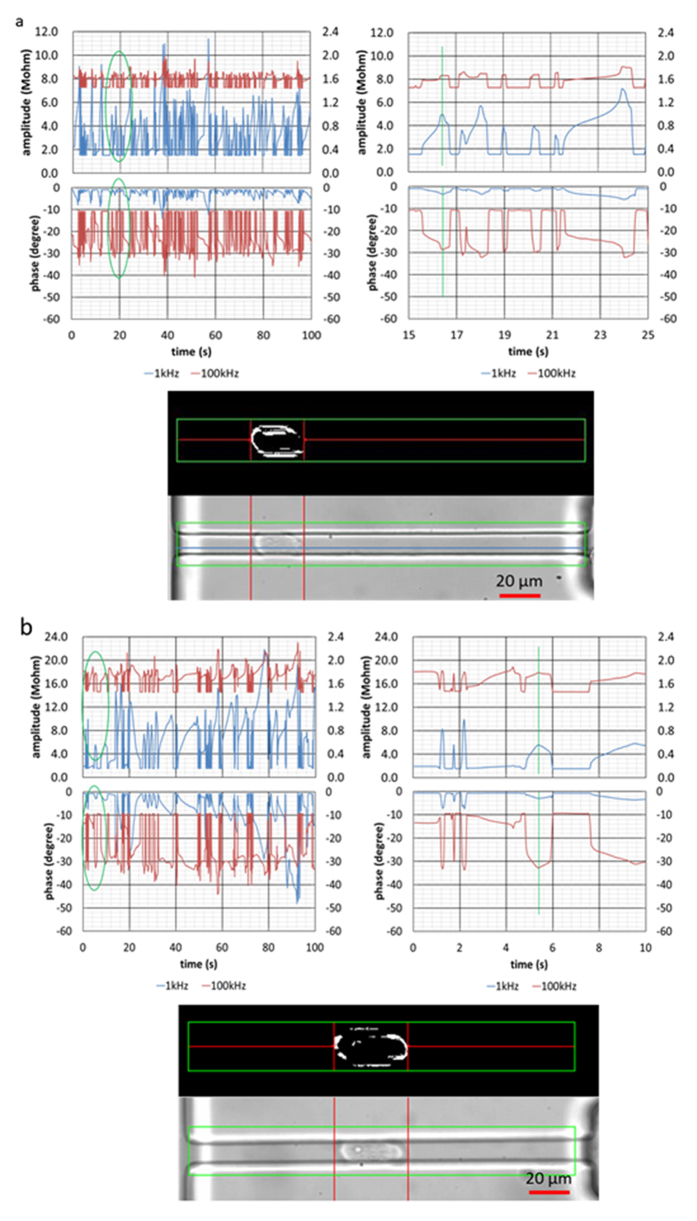
Impedance amplitude and phase measurement at 1 kHz and 100 kHz, simultaneously as well as cellular images squeezing through the constriction channels for A549 (**a**) and H1299 (**b**) based tumor cells, respectively. During the cell squeezing process, there is an amplitude increase and phase decrease for data at both 1 kHz and 100 kHz. Basal impedance amplitudes (no cell entry) at 1 kHz were lower than the values at 100 kHz and there were higher impedance amplitude increases during the cellular squeezing process at 1 kHz than those of 100 kHz. As to the phase data, basal phase values (no cell entry) at 1 kHz were almost zero degree, which were higher than the values at 100 kHz. There were higher phase changes during the cellular squeezing process at 100 kHz than 1 kHz.

Figure 5(a,b) show the impedance amplitude and phase measurement at 1 kHz and 100 kHz, simultaneously as well as images of cellular squeezing through the constriction channels for A549 and H1299 based tumor cells, respectively. During the cell squeezing process, there was an amplitude increase and a phase decrease for impedance data at both 1 kHz and 100 kHz. Basal impedance amplitudes (no cell entry) at 1 kHz were lower than the values at 100 kHz and there were higher impedance amplitude increases during the cellular squeezing process at 1 kHz than those of 100 kHz. As to the phase data, basal phase values (no cell entry) at 1 kHz were almost zero degree, which were higher than the values at 100 kHz. There were higher phase changes during the cellular squeezing process at 100 kHz than 1 kHz.

**Figure 6:**
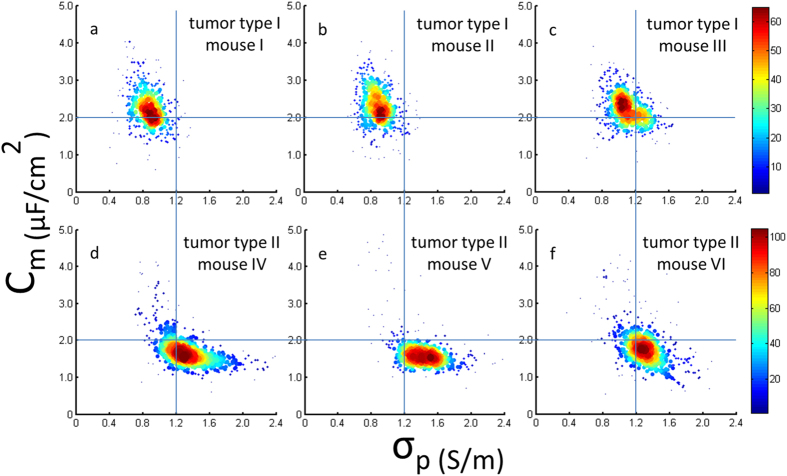
C_m_ and σ_p_ for A549 (a–c) and H1299 (d–f) based tumor samples, respectively. When a cross line (C_m_ = 2.0 μF/cm^2^ and σ_p_ = 1.2 S/m) was drawn to split the scatter plots, electrical properties of A549 and H1299 based tumor samples fall within the upper left domain and the lower right domain, respectively. These results confirm the classification of mouse tumor samples based on C_m_ and σ_p_.

Based on previously developed electrical models^34^,^35^, raw impedance data were translated to C_m_ and σ_p_, two size-independent intrinsic electrical parameters of single cells (see Fig. 6). For A549 based tumor samples, C_m_ and σ_p_ were quantified as 2.25 ± 0.54 μF/cm^2^ and 0.88 ± 0.17 S/m (n_cell_ = 415, Mouse I), 2.30 ± 0.52 μF/cm^2^ and 0.89 ± 0.15 S/m (n_cell_ = 440, Mouse II) as well as 2.22 ± 0.44 μF/cm^2^ and 1.11 ± 0.19 S/m (n_cell_ = 481, Mouse III). Compared to previous data where C_m_ and σ_p_ of pure A549 cell lines were characterized as 2.00 ± 0.60 μF/cm^2^ and 0.73 ± 0.17 S/m (n_cell_ = 487)^6^, for retrieved cells from A549 based tumors, both increases in specific membrane capacitance and cytoplasm conductivity were observed. For H1299 based tumor samples, C_m_ and σ_p_ were quantified as 1.76 ± 0.51 μF/cm^2^ and 1.34 ± 0.30 S/m (n_cell_ = 526, Mouse IV), 1.69 ± 0.53 μF/cm^2^ and 1.42 ± 0.27 S/m (n_cell_ = 410, Mouse V) as well as 1.81 ± 0.57 μF/cm^2^ and 1.30 ± 0.24 S/m (n_cell_ = 506, Mouse VI). When the data were compared to previous data of pure H1299 cell lines (1.63 ± 0.52 μF/cm^2^ and 0.90 ± 0.19 S/m n_cell_ = 489)^6^, only significant increase in cytoplasm conductivity was observed for retrieved H1299 based tumor cells.

Compared to A549 based tumor samples (C_m_ of 2.25 ± 0.50 μF/cm^2^ and σ_p_ of 0.96 ± 0.20 S/m (n_cell_ = 1336, Mouse I, II, III)), H1299 based tumor samples demonstrated lower C_m_ and higher σ_p_ (C_m_ of 1.76 ± 0.54 μF/cm^2^ and σ_p_ of 1.35 ± 0.28 S/m (n_cell_ = 1442, Mouse IV, V, VI)). When a cross line (C_m_ = 2.0 μF/cm^2^ and σ_p_ = 1.2 S/m) was drawn to split the scatter plots, electrical properties of A549 and H1299 based tumor samples fall within the upper left domain and the lower right domain, respectively. These results confirm the classification of mouse tumor samples based on C_m_ and σ_p_.

## Conclusions

In this paper, electrical property differences were located for two types of tumor samples from three mice injected with A549 cells or H1299 cells, respectively, confirming the feasibility of tumor cell classification based on cellular electrical properties. Future work will focus on electrical property characterization of human tumor samples, with the purpose of investigating the feasibility of human tumor sample classification using C_m_ and σ_p_.

## Additional Information

**How to cite this article**: Zhao, Y. *et al*. Single-Cel Electrical Phenotyping Enabling the Classification of Mouse Tumor Samples. *Sci. Rep.* **6**, 19487; doi: 10.1038/srep19487 (2016).
